# Genome-Wide Analysis of DNA Methylation Differences in Muscle and Fat from Monozygotic Twins Discordant for Type 2 Diabetes

**DOI:** 10.1371/journal.pone.0051302

**Published:** 2012-12-10

**Authors:** Rasmus Ribel-Madsen, Mario F. Fraga, Stine Jacobsen, Jette Bork-Jensen, Ester Lara, Vincenzo Calvanese, Agustin F. Fernandez, Martin Friedrichsen, Birgitte F. Vind, Kurt Højlund, Henning Beck-Nielsen, Manel Esteller, Allan Vaag, Pernille Poulsen

**Affiliations:** 1 Steno Diabetes Center, Gentofte, Denmark; 2 Section of Metabolic Genetics, The Novo Nordisk Foundation Center for Basic Metabolic Research, Faculty of Health and Medical Sciences, University of Copenhagen, Copenhagen, Denmark; 3 Department of Immunology and Oncology, National Centre for Biotechnology, CNB-CSIC, Madrid, Spain; 4 Department of Cancer Epigenetics, Spanish National Cancer Research Centre, Madrid, Spain; 5 Cancer Epigenetics Laboratory, Instituto Universitario de Oncologia del Principado de Asturias, HUCA, Universidad de Oviedo, Oviedo, Spain; 6 Department of Endocrinology, Odense University Hospital, Odense, Denmark; 7 Cancer Epigenetics and Biology Program (PEBC), Bellvitge Biomedical Research Institute (IDIBELL), L'Hospitalet de Llobregat, Barcelona, Spain; 8 Department of Endocrinology, Rigshospitalet, Copenhagen, Denmark; 9 Novo Nordisk A/S, Søborg, Denmark; Karolinska Institutet, Sweden

## Abstract

**Background:**

Monozygotic twins discordant for type 2 diabetes constitute an ideal model to study environmental contributions to type 2 diabetic traits. We aimed to examine whether global DNA methylation differences exist in major glucose metabolic tissues from these twins.

**Methodology/Principal Findings:**

Skeletal muscle (*n* = 11 pairs) and subcutaneous adipose tissue (*n* = 5 pairs) biopsies were collected from 53–80 year-old monozygotic twin pairs discordant for type 2 diabetes. DNA methylation was measured by microarrays at 26,850 cytosine-guanine dinucleotide (CpG) sites in the promoters of 14,279 genes. Bisulfite sequencing was applied to validate array data and to quantify methylation of intergenic repetitive DNA sequences. The overall intra-pair variation in DNA methylation was large in repetitive (LINE1, D4Z4 and NBL2) regions compared to gene promoters (standard deviation of intra-pair differences: 10% points vs. 4% points, *P*<0.001). Increased variation of LINE1 sequence methylation was associated with more phenotypic dissimilarity measured as body mass index (r = 0.77, *P* = 0.007) and 2-hour plasma glucose (r = 0.66, *P* = 0.03) whereas the variation in promoter methylation did not associate with phenotypic differences. Validated methylation changes were identified in the promoters of known type 2 diabetes-related genes, including *PPARGC1A* in muscle (13.9±6.2% vs. 9.0±4.5%, *P* = 0.03) and *HNF4A* in adipose tissue (75.2±3.8% vs. 70.5±3.7%, *P*<0.001) which had increased methylation in type 2 diabetic individuals. A hypothesis-free genome-wide exploration of differential methylation without correction for multiple testing identified 789 and 1,458 CpG sites in skeletal muscle and adipose tissue, respectively. These methylation changes only reached some percentage points, and few sites passed correction for multiple testing.

**Conclusions/Significance:**

Our study suggests that likely acquired DNA methylation changes in skeletal muscle or adipose tissue gene promoters are quantitatively small between type 2 diabetic and non-diabetic twins. The importance of methylation changes in candidate genes such as *PPARGC1A* and *HNF4A* should be examined further by replication in larger samples.

## Introduction

Type 2 diabetes (T2D) is a heterogeneous and complex disease resulting from a combination of impaired pancreatic insulin secretion and insulin resistance in tissues such as skeletal muscle, adipose tissue, and liver. The etiology of tissue defects causing T2D is multifactorial. Several gene polymorphisms have been identified [1]–[8], which together with environmental factors such as an adverse fetal environment, aging and obesity increase the risk of T2D [9]–[11].

Phenotypic discordance in monozygotic twins is traditionally attributed to environmental factors distinct for each individual. Therefore, paired analyses of monozygotic twins discordant for a disease phenotype provide an excellent tool for examination of the environmental contribution to the disease in question.

Epigenetics is traditionally referred to as heritable changes in gene expression which are not due to any alteration in DNA sequence. Besides being heritable, epigenetics is altered by environmental factors and hence represents a potential mechanism through which the environment may cause phenotypic variation [12]. Two major classes of epigenetic modifications of the chromatin exist: methylation of cytosine, mostly at cytosine-guanine dinucleotides (CpG)s, and histone modifications, notably acetylations and methylations. The gene promoter regions are rich in CpG sites, forming CpG islands [13], and methylation of these cytosines is thought to silence gene transcription [14], [15]. Histone modifications may result in both activation and silencing of genes [16].

Dietary intervention [17], [18] and exercise [19], [20] have been demonstrated to provoke epigenetic modulation in humans. We previously investigated global differences in methylation of repetitive intergenic DNA sequences and acetylation of histones in young and elderly monozygotic twin pairs. Interestingly, each pair of young twins had essentially similar epigenetic markings, whereas intra-pair differences were substantial in elderly twins, suggesting epigenetic changes to accumulate during life [21]. Therefore, epigenetics has been supposed to play a role in the development of age-related diseases [22]. Data on the role of epigenetics in T2D are still sparse, particularly in the glucose metabolic tissues involved in the pathogenesis of insulin resistant T2D. Increased methylation of *PPARGC1A*, encoding peroxisome proliferator-activated receptor (PPAR)γ co-activator 1α (PGC-1α), was first reported in pancreatic islets from type 2 diabetic individuals [23], and interestingly, similar T2D-related changes were also found in skeletal muscle [24]. In addition, another gene involved in mitochondrial function, *PDK4*, has been shown to have increased methylation in skeletal muscle from T2D patients [25]. Among the known genes associated with genetic risk of T2D some have been identified with DNA methylation differences between type 2 diabetic and healthy individuals including *INS* [26] and *PDX1* [27] in pancreatic islets and *FTO*, *SLC30A8* and *TCF7L2* in leukocytes [28], [29]. Furthermore, a recent epigenome-wide association study of pancreatic islets from type 2 diabetic and non-diabetic deceased individuals found differential methylation in several genes not previously associated with T2D, including *NIBAN* and *CHAC1* which are involved in endoplasmic reticulum stress [30]. Also individuals genetically predisposed to T2D through a family history of T2D have been shown to have altered DNA methylation in skeletal muscle, among others in mitogen-activated protein kinase pathway genes [20].

Applying an epigenome-wide microarray approach in a unique population of elderly monozygotic twins discordant for T2D, the present study aimed to examine whether likely acquired changes in DNA methylation of gene promoters in skeletal muscle and subcutaneous adipose tissue (SAT) associate with T2D. We hypothesized that the genomic identity of the twins would eliminate genetic causes of T2D-related DNA methylation facilitating the identification of acquired changes in a paired study design. On the other hand, the difficulty of obtaining biopsy material from a larger number of the rare T2D-discordant twin pairs is a limitation of the study.

## Results

### Subject Characteristics

**Figure 1:**
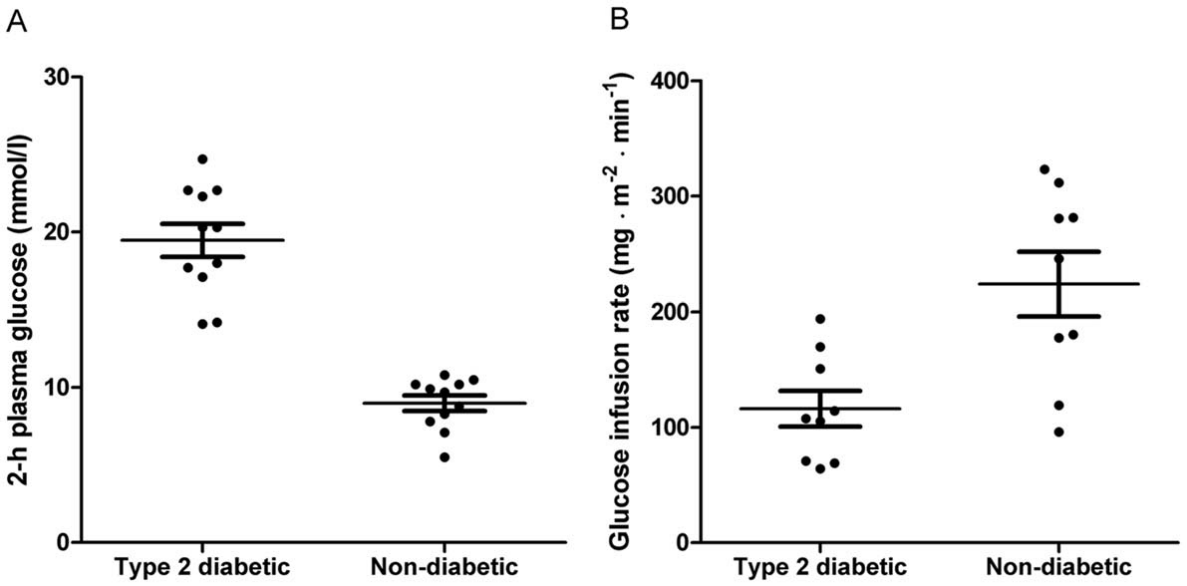
Discordance for oral glucose tolerance and insulin sensitivity in monozygotic twins. A Plasma glucose 120 min after a 75-g oral glucose load in type 2 diabetic and non-diabetic twins (*n* = 12 pairs, *P*<0.001). **B** Glucose infusion rate during a euglycemic-hyperinsulinemic clamp in type 2 diabetic and non-diabetic twins (*n* = 9 pairs, *P* = 0.006). Data are presented as mean±standard error of the mean.

The study population included 12 Danish monozygotic twin pairs discordant for T2D (Table 1). Skeletal muscle and abdominal SAT biopsies were available from 11 and 5 pairs, respectively. The non-diabetic co-twin had normal glucose tolerance in 2 pairs and impaired glucose tolerance in 10 pairs (Figure 1A). Insulin sensitivity, measured as the glucose infusion rate (GIR), was significantly lower in twins with T2D than in their non-diabetic co-twins (Figure 1B). In addition, fasting plasma glucose, blood hemoglobin A1c (HbA1c) and body mass index (BMI) differed significantly between type 2 diabetic and non-diabetic twins (Table 1).

**Table 1:** Characteristics of monozygotic twins discordant for type 2 diabetes.

	Non-diabetic	Type 2diabetic	*P*
*n* (male/female)	12 (6/6)	12 (6/6)	.
Age (years)	68.3±7.7	68.3±7.7	1.0
BMI (kg/m^2^)	30.2±6.3	32.3±6.4	0.02
Fasting plasma glucose(mmol/l)	6.4±0.5	10.5±2.0	<0.001
Fasting serum insulin (pmol/l)	78.9±55.2	95.6±52.3	0.3
Blood hemoglobin A1c (%)	6.0±0.5	7.7±1.4	0.001

Data are shown as mean±standard deviation.

### Global DNA Methylation Patterns

**Figure 2:**
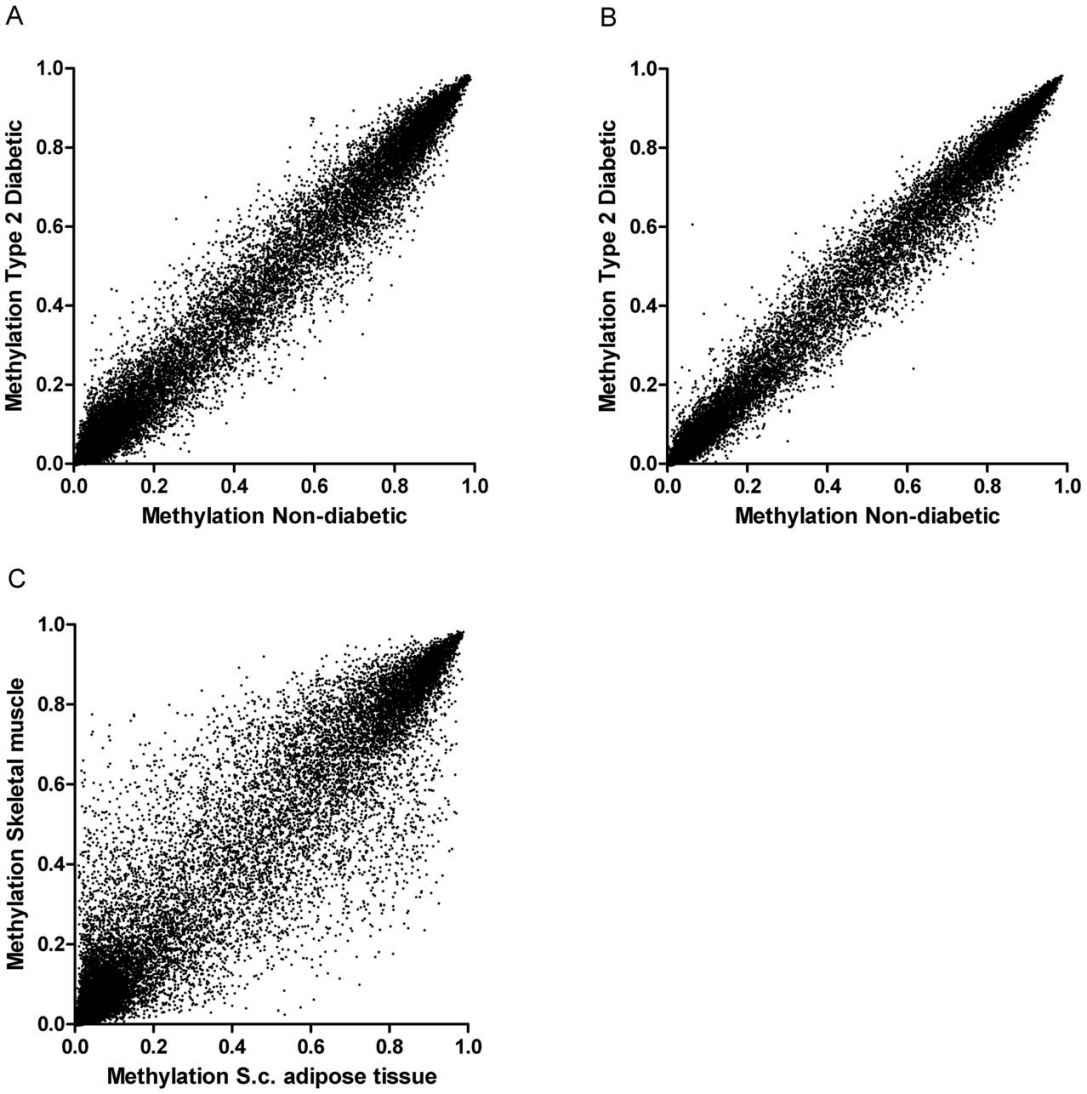
Global gene promoter DNA methylation in monozygotic twins. The DNA methylation was measured as the β-value ranging from 0 (unmethylated) to 1 (completely methylated) at 26,850 CpG sites located in the promoters of 14,279 genes. The plots are shown for a representative twin pair (#3). **A** Skeletal muscle (r = 0.95, *P*<0.001). **B** SAT (r = 0.97, *P*<0.001). **C** Comparison of DNA methylation in SAT and skeletal muscle from the non-diabetic twin.

**Figure 3:**
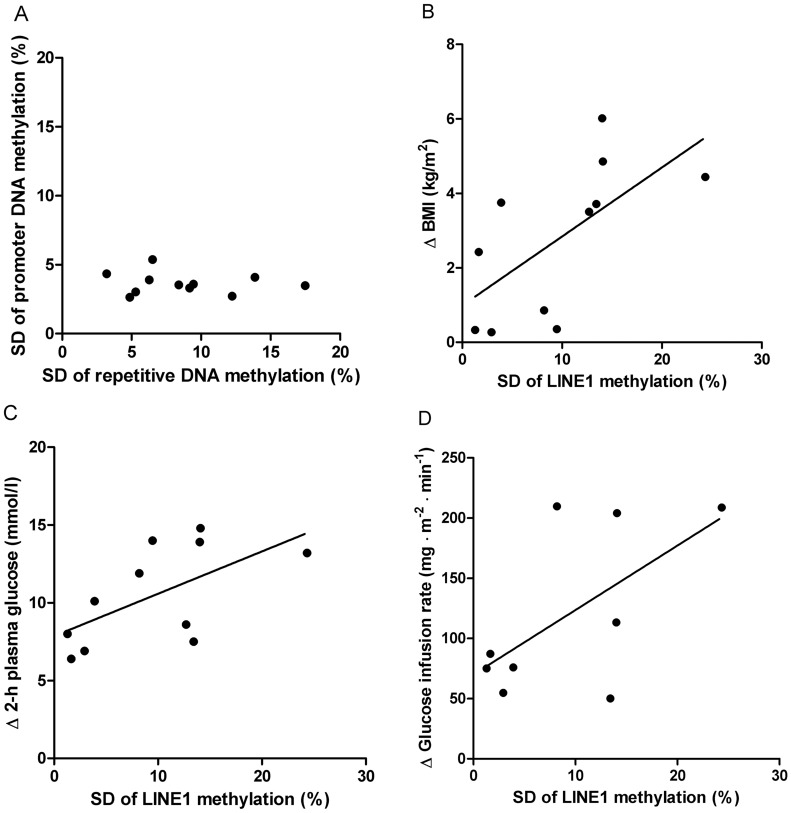
Intra-twin pair variation of DNA methylation in repetitive DNA sequences. The variation is given by the standard deviation (SD) of the absolute methylation differences between monozygotic twins in skeletal muscle. **A** SD of methylation differences on all repetitive versus all promoter CpG sites (r = −0.05, *P* = 0.9). **B** SD of methylation differences on LINE1 repetitive CpG sites versus absolute difference in BMI (r = 0.77, *P* = 0.007). **C** SD of methylation differences on LINE1 repetitive CpG sites versus absolute difference in 2-hour plasma glucose (r = 0.66, *P* = 0.03). **D** SD of methylation differences on LINE1 repetitive CpG sites versus absolute difference in glucose infusion rate (r = 0.52, *P* = 0.2).

In skeletal muscle and SAT, the fractions of low methylated (<25%) CpG sites on the array were 64% and 65%, respectively, whereas the fractions of highly methylated (>75%) sites were 13% and 15%. Twins from a pair were relatively similar regarding DNA methylation in each tissue type (Figure 2A−B), whereas the intra-individual methylation difference between skeletal muscle and SAT was considerable (Figure 2C). The intra-pair methylation differences were slightly larger in skeletal muscle than in SAT. Twin pair was a significant predictor of overall methylation pattern in both skeletal muscle (*P*<0.001) and SAT (*P*<0.001), whereas overall methylation did not differ according to diabetes status (*P*>0.1). However, in both muscle (*P*<0.001) and SAT (*P* = 0.009), twin pair interacted with diabetes status. The variation, expressed as the standard deviation (SD) of absolute intra-pair methylation differences, was significantly greater in repetitive LINE1, D4Z4 and NBL2 DNA sequences than in promoter regions (SD: 10% points vs. 4% points, *P*<0.001), but there was no correlation between the degree of variation in these regions (r = −0.05, *P* = 0.9, Figure 3A). The largest variation was found in methylation of LINE1 CpG sites (SD: 13% points), and it correlated positively with the intra-pair differences in BMI (r = 0.77, *P* = 0.007, Figure 3B) and plasma glucose 2 hours after an oral glucose tolerance test (OGTT, r = 0.66, *P* = 0.03, Figure 3C), but not significantly with the intra-pair difference in GIR (r = 0.52, *P* = 0.2, Figure 3D). The intra-pair variation in D4Z4, NBL2 and promoter regions did not correlate with phenotypic differences in BMI, 2-hour glucose and GIR.

### Differentially Methylated CpG Sites in Type 2 Diabetes

In the 49 known susceptibility genes for mono- or polygenetic T2D represented on the microarray, 136 CpG sites were examined for differential methylation by a candidate approach. Among these sites, 25 sites located in 17 genes (8 sites in muscle and 17 sites in SAT) were differentially methylated between type 2 diabetic and non-diabetic twins (Table 2, Figure S1B,D–E). These candidate genes were *CDKN2A*, *DUSP9*, *HNF4A*, *HHEX*, *KCNQ1*, *KLF11*, *PPARGC1A* and *SLC30A8* in muscle and *ADCY5*, *CAV1*, *CIDEC*, *CDKN2A*, *CDKN2B*, *DUSP9*, *HNF4A*, *IDE*, *IRS1*, *KCNQ1*, *MTNR1B*, *TSPAN8* and *WFS1* in SAT. Two CpG sites in SAT (*CDKN2A* and *HNF4A*) remained statistically significant after permutation correction for multiple testing (*P_adj_* = 0.02; Table 2, Figure S1B,D–E). An explorative analysis of all 26,850 array CpG sites showed that 789 (3%) sites in muscle and 1,458 (5%) sites in SAT were differentially methylated (*P*<0.05) in type 2 diabetic compared with non-diabetic twins before correction for multiple testing. These CpG sites were located in 768 genes in muscle and 1,389 genes in SAT. One CpG site in muscle (*IL8*) and 7 sites in SAT (*ZNF668*, *HSPA2*, *C8orf31*, *CD320*, *SFT2D3*, *TWIST1*, *MYO5A*) remained statistically significant after permutation correction (*P_adj_*<0.001; Table 3). None of the CpG sites in repetitive LINE1, D4Z4 and NBL2 sequences differed significantly in methylation between type 2 diabetic and non-diabetic twins (*P*>0.1).

**Table 2:** Differentially methylated type 2 diabetes susceptibility genes.

Gene	Tissue	CpGs(changed/total)	Probe target ID	Distance to TSS(base pairs)	Type 2diabetic (%)	Non-diabetic(%)	Difference(% points)	*P*	*P_adj_*
**Monogenetic**									
*CAV1*	SAT	1/6	cg27242945	295	2.3±0.5	3.5±0.3	−1.2	0.01	0.7
*CIDEC*	SAT	1/2	cg05684195	168	54.8±3.4	50.0±2.4	4.9	0.003	0.3
*HNF4A*	Skeletal muscle	1/2	cg23834593	−521	84.6±2.8	82.4±2.3	2.2	0.02	0.8
*HNF4A*	SAT	1/2	cg19717150	437	75.2±3.8	70.5±3.7	4.6	0.0003	0.02
*KLF11*	Skeletal muscle	1/2	cg20389709	621	12.7±13.4	9.6±9.6	3.1	0.04	0.9
**Common variety**									
*ADCY5*	SAT	1/2	cg13384396	−285	14.2±2.5	11.7±2.1	2.6	0.03	1.0
*CDKN2A*	Skeletal muscle	1/9	cg07752420		11.7±2.9	13.1±2.3	−1.4	0.04	0.9
*CDKN2A*	SAT	2/9	cg10895543		5.4±0.6	6.0±0.8	−0.6	0.04	1.0
*CDKN2A*	SAT	2/9	cg12840719		12.6±1.9	16.6±2.1	−4.0	0.0003	0.02
*CDKN2B*	SAT	4/10	cg19481686		52.4±12.4	44.7±9.7	7.7	0.02	0.9
*CDKN2B*	SAT	4/10	cg08390209		54.9±8.3	50.0±7.0	4.9	0.008	0.6
*CDKN2B*	SAT	4/10	cg04675937		56.2±10.9	48.2±7.3	8.0	0.03	0.9
*CDKN2B*	SAT	4/10	cg18979223		58.4±8.8	53.5±5.5	4.9	0.04	1.0
*DUSP9*	Skeletal muscle	1/1	cg13915726	321	33.9±12.8	24.6±9.1	9.3	0.01	0.6
*DUSP9*	SAT	1/1	cg13915726	321	20.0±13.4	27.5±14.6	−7.6	0.009	0.6
*HHEX*	Skeletal muscle	1/2	cg11378840	85	6.4±2.8	4.3±1.9	2.0	0.046	0.9
*IDE*	SAT	1/2	cg22812892	247	1.6±0.2	1.3±0.1	0.3	0.03	1.0
*IRS1*	SAT	1/2	cg11620807	122	4.0±0.3	4.1±0.4	−0.1	0.04	1.0
*KCNQ1*	Skeletal muscle	1/23	cg17820828		52.1±11.1	55.5±10.7	−3.4	0.04	0.9
*KCNQ1*	SAT	1/23	cg19728223		18.7±9.0	11.6±3.9	7.2	0.05	1.0
*MTNR1B*	SAT	1/2	cg15842276	−141	36.8±6.9	35.3±6.9	1.5	0.01	0.7
*PPARGC1A*	Skeletal muscle	1/2	cg04893087	−383	13.9±6.2	9.0±4.5	5.0	0.03	0.9
*SLC30A8*	Skeletal muscle	1/2	cg07459489	−174	69.1±5.0	73.9±4.0	−4.8	0.01	0.5
*TSPAN8*	SAT	1/2	cg12965512	−557	46.6±6.6	39.2±7.9	7.3	0.04	1.0
*WFS1*	SAT	1/2	cg09785172	81	1.8±0.3	2.1±0.4	−0.3	0.04	1.0

The microarray included in total 49 type 2 diabetes candidate genes represented by 136 probes. Each CpG site is identified with Illumina probe target ID. The CpG site location is given as the base pair distance to transcription start site (TSS) if available. Data are shown as mean±standard deviation. *P*_adj_-values are corrected for multiple testing (136 tests).

**Table 3:** Differentially methylated genes.

Subcutaneous adipose tissue							
Gene	Target ID	Distance to TSS(base pairs)	Mean type 2 diabetic (%)	Mean non-diabetic (%)	Difference(% points)	*P*	*P_adj_*
*ZNF668*	cg09765256	−3	3.4±0.5	2.6±0.5	0.8	<0.0001	0
*HSPA2*	cg01520924		12.1±1.6	14.8±1.6	−2.7	<0.0001	0
*C8orf31*	cg04612566	27	35.1±6.0	26.9±5.4	8.1	<0.0001	0
*CD320*	cg23963136	266	1.4±0.4	1.9±0.3	−0.5	<0.0001	0
*SFT2D3*	cg11206634	−198	67.9±3.7	74.5±3.6	−6.6	0.0001	0
*TWIST1*	cg22498251	−415	10.6±0.8	12.6±0.8	−1.9	0.0001	0
*MYO5A*	cg23287547	769	2.6±0.4	3.4±0.5	−0.8	0.0001	0
*SULT1A1*	cg18530748	22	6.3±1.2	4.7±1.3	1.5	0.0001	0.1
*PRRX2*	cg04713521	618	4.7±1.1	2.8±1.1	1.9	0.0002	0.9
*TGFA*	cg07004820	479	3.0±0.4	4.2±0.3	−1.2	0.0002	0.9
*WDR8*	cg13501117	60	4.7±0.5	5.4±0.5	−0.6	0.0002	0.9
*ERCC6*	cg14343062	504	3.2±0.7	1.8±0.6	1.4	0.0003	1.0
*HNF4A*	cg19717150	437	75.2±3.8	70.5±3.6	4.6	0.0003	1.0
*ZDHHC8*	cg25650110	98	4.2±0.6	5.6±0.6	−1.4	0.0003	1.0
*CDKN2A*	cg12840719		12.6±1.9	16.6±2.1	−4.0	0.0003	1.0
*GNB5*	cg14120436	67	36.2±6.0	43.0±5.7	−6.8	0.0003	1.0
*CREBL2*	cg09819033	−200	1.7±0.4	2.5±0.3	−0.8	0.0004	1.0
*TMEM79*	cg14500718	−1283	20.7±5.1	28.9±3.9	−8.2	0.0004	1.0
*VGLL1*	cg12384303	−424	58.4±3.6	69.4±4.8	−11.0	0.0005	1.0
*KITLG*	cg18422443	−148	1.9±0.4	2.5±0.5	−0.6	0.0005	1.0
**Skeletal muscle**							
**Gene**	**Target ID**	**Distance to TSS** **(base pairs)**	**Mean T2D (%)**	**Mean non-T2D** **(%)**	**Difference** **(% points)**	***P***	***P_adj_***
*IL8*	cg16468729	190	48.1±7.1	37.3±8.9	10.8	0.0001	0.0003
*GZMB*	cg08766149	131	85.3±2.1	88.3±2.4	−3.0	0.0001	0.4
*MTUS1*	cg22807551	−992	10.4±3.8	7.8±3.7	2.6	0.0002	0.4
*PNOC*	cg03642518	354	55.5±3.5	52.3±4.1	3.2	0.0004	0.9
*PDGFD*	cg07748540	270	4.8±1.3	3.4±0.9	1.5	0.0005	0.9
*KLF2*	cg04898512	−656	89.7±1.2	88.2±1.5	1.5	0.0006	0.9
*ZNF160*	cg12586262	22	13.5±2.8	9.9±1.9	3.6	0.0006	0.9
*PCBP3*	cg23272214	−32	85.8±2.8	88.1±2.5	−2.3	0.0007	1.0
*CDR2*	cg23142935	630	3.3±1.8	2.6±1.6	0.7	0.0010	1.0
*TEKT4*	cg05723825	243	54.6±5.4	59.2±4.9	−4.6	0.0014	1.0
*DHCR24*	cg10073091	604	13.4±2.2	15.3±2.6	−2.0	0.0014	1.0
*ADAM17*	cg24320643	321	14.1±3.3	11.2±3.1	2.9	0.0017	1.0
*SCARA3*	cg26847866	500	52.0±6.8	61.1±5.6	−9.1	0.0017	1.0
*KLHL12*	cg04462209	321	3.7±1.4	2.8±1.4	0.9	0.0017	1.0
*SRPK2*	cg00950418	−122	3.5±1.2	4.5±1.6	−1.1	0.0018	1.0
*AMN*	cg09616556	−348	65.8±2.9	69.8±3.0	−3.9	0.0018	1.0
*FOLR3*	cg07676849	−1106	58.6±6.8	50.9±6.2	7.7	0.0021	1.0
*PTPN1*	cg15864184	−17	10.6±3.7	7.0±3.2	3.5	0.0025	1.0
*CDH13*	cg00806490	295	10.1±2.7	13.1±1.5	−3.0	0.0026	1.0
*PIGR*	cg02105856	23	38.7±2.4	30.6±2.3	8.1	0.0027	1.0

The 20 CpG sites with the lowest *P*-values for the intra-pair methylation difference between type 2 diabetic and non-diabetic twins are shown for subcutaneous adipose tissue and skeletal muscle. The total number of differentially methylated CpG sites, without correction for multiple testing, was 1,458 in subcutaneous adipose tissue and 789 in skeletal muscle. Each CpG site is identified with Illumina probe target ID. The CpG site location is given as the base pair distance to transcription start site (TSS) if available. Data are shown as mean±standard deviation. *P*_adj_-values are corrected for multiple testing (26,850 tests).

### Validation of Array Data

We have recently validated the Illumina DNA methylation array’s ability to detect methylation differences of approximately 10% points in paired skeletal muscle biopsies from an overfeeding intervention study [18]. In the present study, five genes (three in SAT and two in muscle) with larger absolute methylation differences (>4% points) were selected for validation of the microarray data in three twin pairs. Bisulfite sequencing (BS) was used in SAT for *MCF2*, *HNF4A* and *FAP*, and bisulfite pyrosequencing (PBS) was used in muscle for *PPARGC1A* and *SLC30A8*. The methylation differences for these genes were successfully validated (Figure S1).

### Pathway Analyses

Ingenuity Pathway Analysis software was used to identify molecular pathways with a significant proportion of genes having CpG sites differentially methylated between type 2 diabetic and non-diabetic twins. The analysis was done on genes with at least one differentially methylated GpC site based on the uncorrected *P*-values. Nine muscle pathways were identified, including inflammatory (hepatic fibrosis and IL-6), lipid metabolic (PPARα, PPARγ and sphingolipids) and carbohydrate metabolic (pyruvate and propanoate) pathways (Table S2). In SAT, one carbohydrate metabolism and one circadian rhythm signaling pathway had a significant fraction of genes with methylation changes (Table S2).

## Discussion

We have previously used a population of twelve monozygotic twin pairs discordant for T2D to recognize a considerable non-genetic contribution to glucose metabolic disturbances in genetically identical individuals [31]. In the present study, we used a similar twin sample for the investigation of genome-wide common acquired changes in gene promoter DNA methylation in the primary insulin responsive tissues, skeletal muscle and SAT. We found that absolute intra-twin pair methylation differences were relatively small, and most of them did not link significantly to T2D after correction for multiple testing. The findings altogether suggest a modest contribution of acquired DNA methylation differences in skeletal muscle or SAT to the non-genetic component of T2D.

It has been demonstrated in a prospective study that DNA methylation changes occur over time, suggesting an influence by environmental or stochastic events [12]. A study of monozygotic and dizygotic twins has provided evidence that such epigenetic changes are more pronounced in non-CpG island than in CpG island DNA regions [32]. In addition, a clustering of DNA methylation changes in families indicated that the susceptibility to a given epigenetic change may have a genetic component [12]. We hypothesized that the use of genetically identical twins with its inherent correction for the genetic influence on both disease phenotype and DNA methylation would represent the ideal design to evaluate acquired DNA methylation changes associated with T2D.

We investigated the methylation percentage of 26,850 gene promoter CpG sites in skeletal muscle and SAT and found that the majority of these sites were clustered into two groups with either a low or a high methylation percentage. This indicates a high concordance of methylation for the DNA copies present in a biopsy. Furthermore, co-twins showed a greater degree of similarity in gene promoter methylation than unrelated individuals. The overall promoter methylation did not depend on T2D status alone, but interestingly it was significantly associated with the interaction between T2D status and twin pair. This suggests that, within each pair, T2D-related DNA methylation differences might exist.

The intra-individual DNA methylation pattern in gene promoters differed vastly between tissues, proposing that DNA methylation changes associated with tissue development greatly exceed changes occurring in the finally differentiated tissue. Thus, our data support the notion of a major role of genetics as well as tissue specificity in determining the methylation pattern of gene promoters in adults. The intra-pair methylation differences were slightly larger in skeletal muscle than in SAT. This phenomenon could reflect a greater environmental influence on DNA methylation in skeletal muscle, but could also be due to a greater diversity of cell types in this tissue.

In contrast to gene promoters, we found a considerable intra-pair variation in methylation of genome-wide repetitive DNA sequences. We have previously demonstrated that differences in DNA methylation of non-coding regions are more prominent in elderly monozygotic twins who have spent less of their lives together than those having shared the environment for a longer period [21]. A novel finding of our present study is that the variation in methylation levels of repetitive LINE1 DNA, which makes up 17% of the human genome [33], was largest in twin pairs being different in BMI and 2-hour plasma glucose. This result provides evidence that epigenetic variation reflects the degree of dissimilarity between phenotypes of monozygotic twins. However, in agreement with a recent study of human pancreatic islet cells [30] we found no specific methylation changes in repetitive DNA sequences between type 2 diabetic and non-diabetic individuals.

Despite being relatively small, a number of the intra-pair methylation differences in gene promoters identified by the microarrays were successfully validated by two different bisulfite sequencing methods. The validation additionally showed that CpG sites close to the array site were likely to be co-methylated supporting previous observations [14], [17]. Although this technical validation does not exclude the modest biological differences to have occurred by chance, it supports the sensitivity of the microarray to detect methylation differences down to a few percentage points. Previous studies have reported large methylation differences between cancer cells and normal cells [15], which may among others be attributed to the fact that the tumor comprises a clone of abnormal cells. In the, by comparison, healthier and non-dividing muscle and SAT tissues of type 2 diabetic individuals, it could be speculated that environmentally determined methylation changes may take place in only some cells. Recent studies of *IGF2* [34], *HNF4A* [35] and *PPARGC1A* [17], [18] methylation in blood or skeletal muscle from individuals exposed to an adverse intrauterine environment showed that methylation differences between exposed and unexposed individuals were of a similar size to those found in our study. This was also the case for reported T2D-associated methylation differences of *PPARGC1A* [23], *INS* [26] and *PDK4* [25] in pancreatic islets or skeletal muscle. mRNA expression of these genes was altered in T2D and in some cases correlated with DNA methylation [23], [26]. However, epigenome-wide DNA methylation profiling studies in pancreatic islets [30] and skeletal muscle [18] with expression analysis of a fraction of the differentially methylated genes show that the relationship between small DNA methylation differences and gene expression is complicated. Several factors such as location of the specific DNA methylation site in relation to gene regulatory regions and co-existence of other regulatory mechanisms complicate the study of smaller methylation differences’ biological impact.

Most known T2D susceptibility gene polymorphisms associate primarily with β-cell dysfunction making the endocrine pancreas the likely tissue for T2D-associated DNA methylation changes. A recent genome-wide study of T2D-associated DNA methylation differences in a small sample of pancreatic islet biopsies identified many differentially methylated genes in T2D, but none of the known susceptibility genes were among these [30]. *IRS1* [8] and *PPARGC1A* [2] belong to the small group of T2D susceptibility genes which might mediate their effects through insulin resistance and therefore be relevant in peripheral tissues such as SAT and skeletal muscle. When the known T2D susceptibility genes represented on the array were specifically evaluated, we found that *ADCY5*, *CAV1*, *CIDEC*, *CDKN2A*, *CDKN2B*, *DUSP9*, *HNF4A*, *IDE*, *IRS1*, *KCNQ1*, *MTNR1B*, *TSPAN8* and *WFS1* had at least one CpG site differentially methylated between type 2 diabetic and non-diabetic twins in SAT. In skeletal muscle, CpG sites in *CDKN2A*, *DUSP9*, *HNF4A*, *HHEX*, *KCNQ1*, *KLF11*, *PPARGC1A* and *SLC30A8* were differentially methylated. However, only the maturity-onset diabetes of the young (MODY) gene *HNF4A* [36] and the T2D gene *CDKN2A* [37] in SAT had significant permutation adjusted *P*-values. Importantly, the absolute methylation differences identified were smaller than those predicted to be found at 80% power, particularly in the adipose tissue samples obtained from only five twin pairs. This finding contrasts the methylation differences of ∼20–30% points identified in a similarly sized sample of pancreatic islets from type 2 diabetic and non-diabetic individuals [30] and could be interpreted as the endocrine pancreas being indeed the tissue with most pathological changes in T2D. In addition to the statistical limitation, some of the differences on low-methylated CpG sites such as in *IRS1* had absolute methylation differences that are probably too small to be detected reliably by the DNA methylation microarray. The finding of increased *HNF4A* methylation in tissues from type 2 diabetic individuals is interesting considering the recent similar result in umbilical cord blood leukocytes from newborns with intrauterine growth restriction [35]. Thus, methylation of this gene could represent a mechanism linking early intrauterine programming to development of T2D later in life. Even though the increased methylation of *PPARGC1A* in type 2 diabetic individuals was not significant after correction for multiple testing, the result corresponds well with previous findings in β-cells [23] and skeletal muscle from type 2 diabetic patients [24]. To this end, we recently reported elevated methylation of the *PPARGC1A* promoter in skeletal muscle from lean and otherwise healthy young men born with low birth weight. Moreover, five days of high-fat, high-calorie feeding increased *PPARGC1A* promoter methylation in healthy men with normal birth weight [17]. Thus, increased methylation of *PPARGC1A* in skeletal muscle seems to be a consistent finding in patients with overt T2D as well as in individuals at risk of developing T2D. The recently discovered T2D susceptibility gene, *DUSP9*, is another gene which could be important to insulin resistance. It encodes mitogen-activated kinase phosphatase-4 which counteracts stress-induced insulin resistance [38]. Interestingly, this gene had methylation changes of 8−9% points dependent on tissue which was among the largest found on the array.

The combination of small absolute methylation differences and a relatively small sample size made it difficult to identify statistically significant associations between methylation changes and T2D by an explorative approach. We found 789 and 1,458 of 26,850 CpG sites which were differentially methylated in skeletal muscle and SAT, respectively. These approximately 5% of the CpG sites investigated could be chance findings, and the fact that only a few CpG sites remained significant after correction for multiple testing contributes to this interpretation of the results. Genes for which the permutation corrected *P*-values were significant included *IL8* in muscle and *TWIST1* in SAT. The inflammatory cytokine IL-8 is expressed in among others skeletal muscle in response to exercise [39], and the transcription factor Twist1 regulates expression of inflammatory cytokines in adipocytes [40].

Molecular pathway analyses were used to examine whether the 768 muscle and 1,458 SAT genes which, based on the explorative analysis, were most likely to have DNA methylation changes belonged to specific functional pathways. Since the methylation differences in the vast majority of these genes were not significant after correction for multiple testing the results should be interpreted cautiously. We found the genes in muscle to be predominantly involved in inflammation, lipid metabolism, and carbohydrate metabolism. Low-grade inflammation is considered to be an important mechanism in insulin resistance [41], and the lipid metabolic regulators PPARα, PPARγ, as well as PGC-1α have been shown to play a role in the regulation of insulin sensitivity [42]–[44]. In SAT, carbohydrate metabolism and circadian rhythm signaling pathways had a significant fraction of genes differentially methylated between type 2 diabetic and non-diabetic individuals. Recent studies have shown that genetic variation in *MTNR1B* and *CRY2*, involved in circadian signaling, associate with T2D or related metabolic traits [3], [45]. Interestingly, both of these genes were differentially methylated in SAT from type 2 diabetic and non-diabetic twins.

In conclusion, common skeletal muscle and adipose tissue gene promoter DNA methylation differences between monozygotic twins discordant for T2D were small, but a number of the differences were found in known T2D-related candidate genes. Larger study groups, preferably with longitudinal sample collection and methylation analysis of the entire promoter are needed to replicate the findings in these genes.

## Research Design and Methods

### Study Participants

Twelve 53–80 year-old monozygotic twin pairs discordant for T2D were recruited through the Danish Twin Registry, University of Southern Denmark. Six pairs had participated in a previous study [46] where discordance for T2D was recognized based on an OGTT. These pairs were reexamined for the present study. The additional six pairs were recruited based on information from the Twin Registry about known T2D. All study participants had provided written informed consent, and the study was approved by the regional Ethical Committee (Southern Denmark, http://komite.regionsyddanmark.dk) and conducted in accordance with the principles of the Helsinki Declaration.

### Clinical Examination

Height and weight were measured in light weight clothes for calculation of BMI. Discordance for T2D was verified by a 75-g OGTT. Insulin sensitivity was measured in a subpopulation (*n* = 9 pairs) by a euglycemic-hyperinsulinemic clamp (40 mU m^−2^ min^−1^) and expressed as the mean GIR during the last 30 min of the clamp period.

### Biopsies

Biopsies were excised under local anesthesia (lidocaine) from the vastus lateralis muscle (*n* = 11 pairs) and from abdominal SAT (*n* = 5 pairs) using a Bergström needle with suction applied. The tissue samples were frozen immediately in liquid nitrogen and stored at −80°C until processed further.

### DNA Extraction

Genomic DNA was extracted from the biopsies using a DNeasy Blood & Tissue Kit (Qiagen, Hilden, Germany), and DNA concentrations were determined by a Quant-IT PicoGreen dsDNA Kit (Invitrogen, Carlsbad, CA).

### DNA Methylation Microarrays

Methylation was assessed at 27,578 CpG sites primarily located close to the transcription start site (TSS) in the promoters of 14,475 genes using 12-sample Infinium HumanMethylation27 Bead Chips (Illumina, San Diego, CA). 600 ng DNA was bisulfite-treated to deaminate unmethylated cytosines to uracil using an EZ DNA Methylation Kit (Zymo Research, Orange, CA). The array was scanned by a BeadArray Reader (Illumina), and intensity data analyzed using GenomeStudio software (version 2011.1, Illumina). Internal array controls verified the hybridization, staining and washing procedures. The methylation status on each CpG site was expressed as the β-value which is the ratio between fluorescent signal from converted and preserved sequence bead types. DNA samples from twin pairs were analyzed on the same bead chip to eliminate a possible influence of batch effect in the subsequent paired statistical analyses. Due to reruns four pairs of muscle samples were separated on different chips. Batch effect in the data set was examined for by unsupervised cluster analysis using MeV software (version 4.5, available at http://www.tm4.org) and scatter plots of principal components 1 and 2. Since only minimal batch effect was found compared to clustering according to twin pair and sex, no further normalization of the β-values was performed. β-values for probes where intensity did not exceed the background level (*P*≥0.05) were omitted (in average 54 β-values per muscle sample and 23 β-values per SAT sample). All probes were examined for unique genome alignment and for SNPs affecting the CpG site using NCBI human genome build 36 FASTA files and custom PERL (version 5.10.1, available at http://www.perl.org) scripts. For the analysis of unique alignment, two “bisulfite-treated” reference genomes were constructed replacing cytosine by thymine as forward strand reference, and replacing guanine by adenine as reverse strand reference. The unmethylated bead type probe sequences were compared to these reference genomes using the BLAT algorithm [47]. For the SNP analysis, Illumina’s annotation data were used to mark each target region in a reference genome containing dbSNP build 130 information. All corresponding SNP-masked sequences were extracted and screened for SNPs. 494 probes were excluded due to multiple alignments, and additionally 234 were excluded due to SNP in the CpG site. The microarray data have been deposited in the Gene Expression Omnibus (GEO) database (http://www.ncbi.nlm.nih.gov/geo, accession number GSE38291) complying with the Minimum Information About a Microarray Experiment (MIAME) guidelines.

### Validation and Methylation of Repetitive DNA

Array data were validated in three genes in SAT by BS and in two genes in skeletal muscle by BPS. DNA methylation of LINE1 (interspersed repeat with 8 CpG sites), D4Z4 (tandem repeat with 9 CpG sites) and NBL2 (tandem repeat with 8 CpG sites) sequences was measured by BPS in the muscle samples. The repetitive distribution of these sequences means that the DNA methylation measured is a genome-wide average value.

#### Bisulfite sequencing

1 µg DNA was bisulfite-treated as described previously [48]. Oligonucleotide primers (Sigma-Aldrich, St. Louis, MO) were designed using Methyl Primer Express Software (Applied Biosystems, Foster City, CA) to make the PCR amplicons cover approximately five CpG sites, including the site analyzed on the array (Table S1). The PCR product was separated on a 2% agarose gel, and specific DNA bands were cut and purified by a GFX PCR DNA and Gel Band Purification Kit (GE Healthcare, Buckinghamshire, UK). The specific amplicons were cloned in *E. coli* using the pGEM-T vector system (Promega, Madison, WI). Twelve positive colonies were collected, and plasmid DNA was purified by a Perfectprep Plasmid 96 Vac Kit (Eppendorf, Hamburg, Germany). The plasmid DNA insert was finally sequenced on an ABI 3100 system (Applied Biosystems). The number of methylated and unmethylated clones was counted, and the methylation percentage calculated as the average methylation of all CpG sites in the amplicon.

#### Bisulfite pyrosequencing

BPS was performed on bisulfite-treated DNA using a PyroMark MD pyrosequencing system (Biotage, Uppsala, Sweden). Specific pyrosequencing primers were designed to amplify as many CpGs as conditions permitted (1 to 9 sites) using Assay Design Software (version 1.0.6, Biotage, Table S1). The cytosine methylation percentage was evaluated with the Pyro Q-CpG program (version 1.0.9, Biotage).

### Statistical Methods

The statistical analyses were performed using R software (version 2.11.1, available at http://www.r-project.org). β-values are shown in percentages and differences given in percentage point change (type 2 diabetic twin − non-diabetic twin). The differences were examined for normality using the Shapiro-Wilk normality test. Only 6% of the CpG sites in adipose tissue and 9% of the CpG sites in skeletal muscle had *P*-values <0.05 suggestive of non-normally distributed differences, and therefore parametric statistics were applied.

#### Analysis of twin pair similarity

An ANOVA including methylation of all microarray CpG sites as response variable and twin pair, diabetes status and their interaction as explanatory variables was used to evaluate the effect of twin pairs on the overall DNA methylation similarity. For correlation analyses the intra-pair variation in promoter or repetitive DNA methylation was expressed as the SD for the absolute differences.

#### Analysis of differential methylation by candidate gene approach

All monogenic and confirmed common variety T2D susceptibility genes reviewed by O’Rahilly [1] were examined for differential methylation if represented on the array. In addition, *KCNQ1* [6] and *KLF11* [7] as well as the newly discovered genes, *ADCY5*, *GCK*, *GCKR, PROX1*, *DUSP9*, *HMGA2*, *KLF14*, *TP53INP1* and *RBMS1* [3]–[5] were included. *PPARGC1A* [2] was included due to an a priori hypothesis of altered DNA methylation in type 2 diabetic individuals [23], [24]. Thus, the genes analyzed were *ABCC8*, *ADAMTS9*, *ADCY5, AGPAT2*, *AKT2*, *BSCL2*, *CAMK1D*, *CAV1*, *CDC123*, *CDKAL1*, *CDKN2A*, *CDKN2B*, *CEL*, *CIDEC*, *DUSP9*, *HHEX*, *HMGA2*, *HNF1A*, *HNF1B*, *HNF4A*, *GCK*, *GCKR*, *IDE*, *IGF2BP2*, *INS*, *INSR*, *IRS1*, *JAZF1*, *KCNJ11*, *KCNQ1*, *KIF11*, *KLF11*, *KLF14*, *LGR5*, *LMNA*, *MTNR1B*, *NEUROD1*, *NOTCH2*, *PDX1*, *PPARG*, *PPARGC1A*, *PROX1*, *RBMS1*, *SLC30A8*, *TBC1D4*, *TCF7L2*, *TP53INP1*, *TSPAN8*, and *WFS1*. These 49 genes were represented by 136 probes. Comparisons between type 2 diabetic and non-diabetic twins were performed by paired *t*-tests. Two-sided *P*<0.05 was considered statistically significant, and the *P*-values were corrected for multiple testing, *P_adj_*, using the Westfall-Young resampling method [49]. 10,000 simulations with permutations of the sample labels were used to sample the null distribution.

#### Analysis of differential methylation by explorative approach

Comparisons between type 2 diabetic and non-diabetic twins were performed for all 26,850 array CpG sites by paired *t*-tests, and the *P*-values were corrected for multiple testing as described above.

#### Statistical power calculations

The study population size was limited to the maximal number of Danish T2D discordant monozygotic twin pairs that could be recruited in a 10-year period. From previous studies of *PPARGC1A*, DNA methylation differences of ∼5% points with ∼5% points SD have been identified [17], [23]. Given the 11 pairs of muscle biopsies and a fixed SD of 5% points the study allowed detecting a 5% point methylation difference in a single paired *t*-test at 80% power. The size of the minimal detectable differences would increase to 10% in the candidate gene analysis (136 *t*-tests) and 17% in the genome-wide analysis (26,850 *t*-tests) when applying Bonferroni correction. For the only 5 pairs of adipose tissue biopsies the similar minimal detectable differences would be 8%, 31% and 117% for single CpG site analysis, candidate gene study and hypothesis-free approach, respectively.

#### Validation of methylation array data

Differences in DNA methylation levels between three pairs of type 2 diabetic and non-diabetic twins obtained by BS or BPS were analyzed by paired *t*-test. Based on the a priori hypothesis of replicating array findings one-sided *P*<0.05 was considered statistically significant.

#### Molecular pathway analyses

Differentially methylated genes identified by the explorative approach, without correction for multiple testing, were included in Ingenuity Pathway Analyses (version 7.5, Ingenuity Systems, Redwood City, CA). This software recognized the pathways from the Ingenuity Pathways Analysis library of canonical pathways (81 metabolic and 283 signaling) which contained a significant fraction of differentially methylated genes. Only genes represented on the array were used for the reference pathways. Fischer’s exact test was used to calculate the probability that the association between the differentially methylated genes and the canonical pathway was explained by chance. These *P*-values were not corrected for multiple testing.

## Supporting Information

Figure S1 **Validation of array data.** The average DNA methylation in the gene promoter area surrounding the array site measured by bisulfite sequencing (BS) or bisulfite pyrosequencing (BPS) is indicated in type 2 diabetic (black bars) and non-diabetic (white bars) twins. Three twin pairs were included in the validations. One-sided *P*-values are shown for the difference between type 2 diabetic and non-diabetic twins measured by BS or BPS. Gene diagrams, including the number of methylated (black) and unmethylated (white) clones for each CpG site in the amplicon, are shown for BS results. **A** *MCF2* +226 base pairs from transcription start site (TSS) in subcutaneous adipose tissue (*P*<0.001), **B** *HNF4A* −521 base pairs from TSS in subcutaneous adipose tissue (*P* = 0.04), **C** *FAP* +80 base pairs from TSS in subcutaneous adipose tissue (*P*<0.001), **D** *PPARGC1A* −383 base pairs from TSS in skeletal muscle (*P* = 0.006), **E** *SLC30A8* −174 base pairs from TSS in skeletal muscle (*P* = 0.002). Data are presented as mean±standard error of the mean.
(PDF)

Table S1 Primer sequences.
(DOC)

Table S2 **Molecular pathways with a significant fraction of differentially methylated genes.** No overlap denotes the fraction of genes in each pathway which was not represented on the array. The direction of methylation change in type 2 diabetic versus non-diabetic twins is indicated by arrows (↑: increased, ↓: decreased).
(DOC)
