# Supplementary figures and images for: Genome-Wide Analysis of DNA Methylation Differences in Muscle and Fat from Monozygotic Twins Discordant for Type 2 Diabetes
Source: PLoS One. 2012 Dec 10;7(12):e51302. doi: 10.1371/journal.pone.0051302 (PMC3519577; doi:10.1371/journal.pone.0051302)

**A**

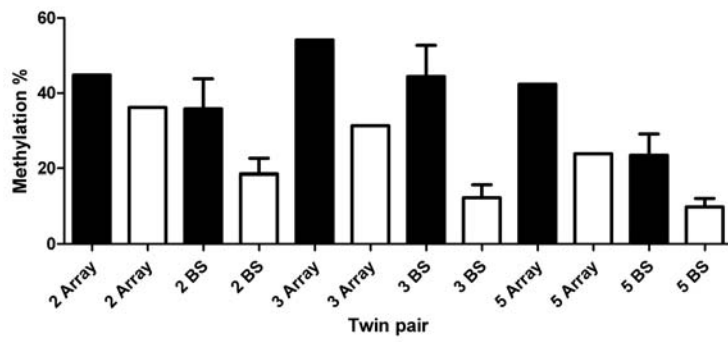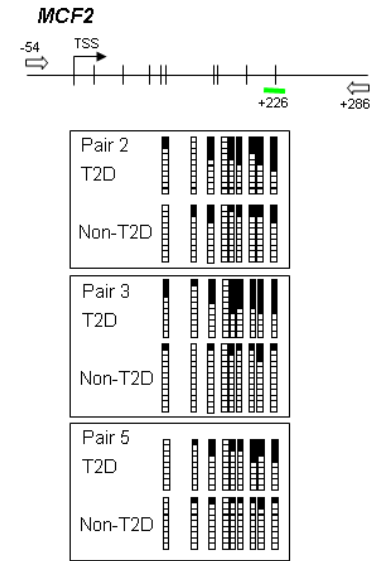

**B**

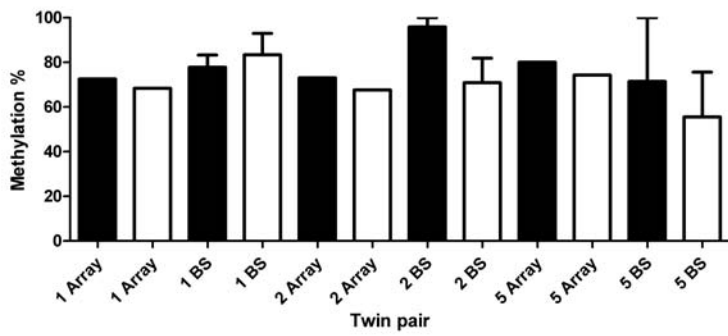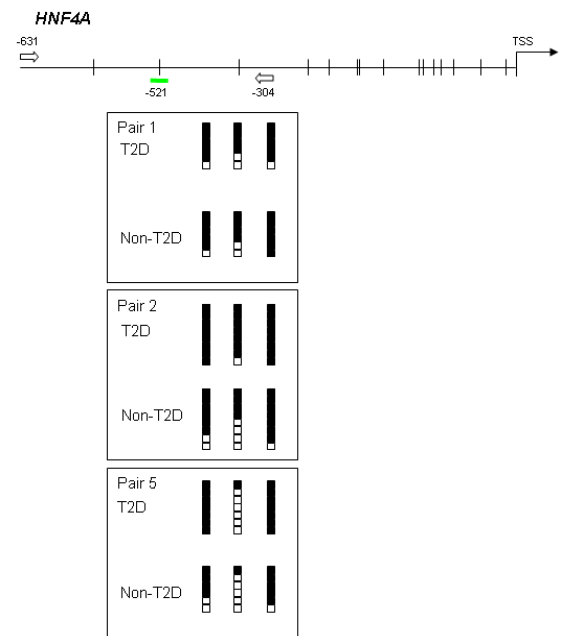

**C**

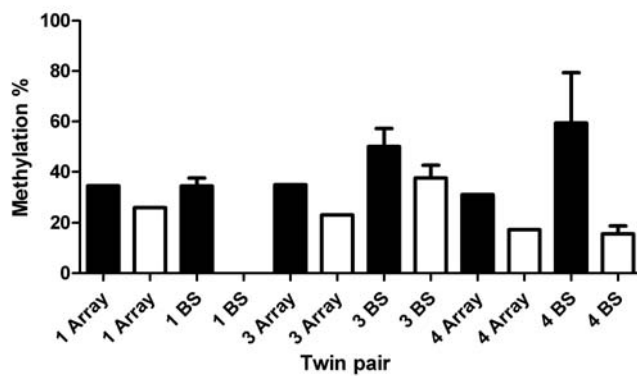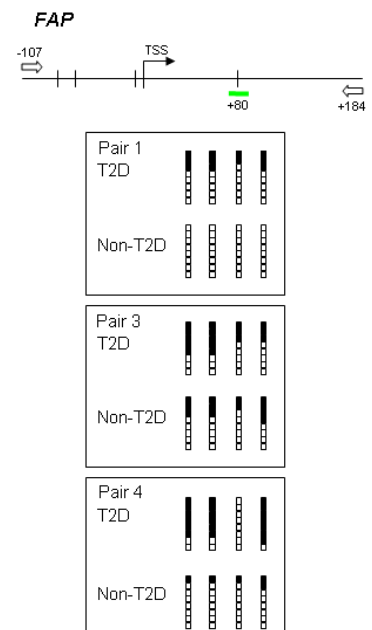

**D**

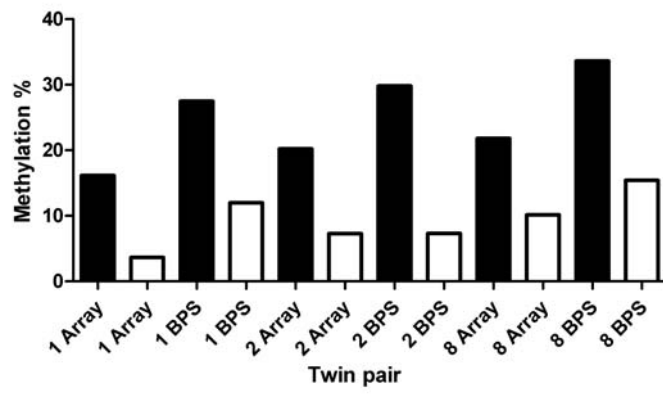

**E**

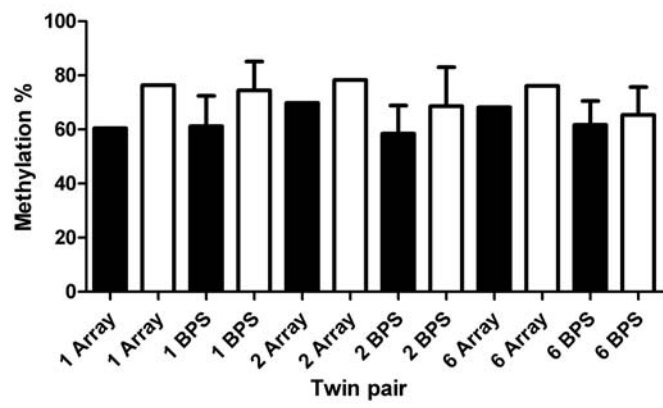

Supplement: Figure S1 — Validation of array data. The average DNA methylation in the gene promoter area surrounding the array site measured by bisulfite sequencing (BS) or bisulfite pyrosequencing (BPS) is indicated in type 2 diabetic (black bars) and non-diabetic (white bars) twins. Three twin pairs were included in the validations. One-sided P-values are shown for the difference between type 2 diabetic and non-diabetic twins measured by BS or BPS. Gene diagrams, including the number of methylated (black) and unmethylated (white) clones for each CpG site in the amplicon, are shown for BS results. A MCF2 +226 base pairs from transcription start site (TSS) in subcutaneous adipose tissue (P<0.001), B HNF4A −521 base pairs from TSS in subcutaneous adipose tissue (P = 0.04), C FAP +80 base pairs from TSS in subcutaneous adipose tissue (P<0.001), D PPARGC1A −383 base pairs from TSS in skeletal muscle (P = 0.006), E SLC30A8 −174 base pairs from TSS in skeletal muscle (P = 0.002). Data are presented as mean±standard error of the mean. (PDF) [file pone.0051302.s001.pdf]
