# Supplementary material for: Genome-Wide Analysis of DNA Methylation Differences in Muscle and Fat from Monozygotic Twins Discordant for Type 2 Diabetes
Source: PLoS One. 2012 Dec 10;7(12):e51302. doi: 10.1371/journal.pone.0051302 (PMC3519577; doi:10.1371/journal.pone.0051302)
Supplement: Table S1 — Primer sequences. (DOC) [file pone.0051302.s002.doc]

| **Gene / Sequence** | **Type** | **Primers** | **Annealing temp. (Tm)** |
| --- | --- | --- | --- |
| *FAP* | BS | Forward: GGAAGTTGAAGTTAGGATAAGG  Reverse: AACCCAATAACTTACTAAAATAATCC | 57 ºC |
| *HNF4A* | BS | Forward: TGTTAGATGAAAGGAAGATAATATTT  Reverse: TATATAAAAACATCAACCCCC | 57 ºC |
| *MCF2* | BS | Forward: AAGGGAAGGATTTGTTAGTTGA  Reverse: ACACCATACTACAACATCCCAA | 60 ºC |
| *PPARGC1A* | BPS | Forward: TGAGTTTGAGTTTATTTGGAGATT  Reverse: Bio-CAAATAACAAAACTCCCTATTTCA  Seq : TGGAGATTTTAGAATTAAAG | 57 ºC |
| *SLC30A8* | BPS | Forward: GAGGGAGAGAAAAAGAAAGA  Reverse: Bio-AACTACTTTTCTAAAAAAACTTACAA  Seq: AGAAAGAATTAGTTTAGTGT | 53 ºC |
| LINE1 | BPS | Forward: TTTTTTGAGTTAGGTGTGGG  Reverse: Bio-TCTCACTAAAAAATACCAAACAA  Seq : GGGTGGGAGTGAT | 56 ºC |
| NBL2 | BPS | Forward: AGTAGTTGGTGTTAATGTGTGTT  Reverse: Bio-AAACCTCTTTACTCCTCTAATAAAC  Seq: TGGTGTTAATGTGTGTTAT | 55 ºC |
| D4Z4 | BPS | Forward: GGTGGTTYGGGGTAGGG  Reverse: Bio-CCCAAAAAAAAATAACAATTCTC  Seq: GGGAATATTTGGTTGGTTA | 60 ºC |
