# Supplementary material for: Genome-Wide Analysis of DNA Methylation Differences in Muscle and Fat from Monozygotic Twins Discordant for Type 2 Diabetes
Source: PLoS One. 2012 Dec 10;7(12):e51302. doi: 10.1371/journal.pone.0051302 (PMC3519577; doi:10.1371/journal.pone.0051302)
Supplement: Table S2 — Molecular pathways with a significant fraction of differentially methylated genes. No overlap denotes the fraction of genes in each pathway which was not represented on the array. The direction of methylation change in type 2 diabetic versus non-diabetic twins is indicated by arrows (↑: increased, ↓: decreased). (DOC) [file pone.0051302.s003.doc]

| **Skeletal muscle** | | | |
| --- | --- | --- | --- |
| **Pathway** | **Regulation in type 2 diabetes** | **Genes** | ***P*** |
| BMP signaling pathway | Decreased 7/80 (9%). No change 56/80 (70%). Increased 3/80 (4%). No overlap 14/80 (18%). | *MAGED1*↓, *BMP4*↓, *BMP8B*↑, *RRAS*↑, *CREBBP*↓, *SMAD6*↓, *PRKAG2*↑, *KRAS*↓, *MAPK12*↓, *BMP8A*↓ | 0.003 |
| Glycosaminoglycan Degradation | Decreased 4/61 (7%). No change 22/61 (36%). Increased 1/61 (2%). No overlap 34/61 (56%). | *HYAL3*↓, *SULF1*↑, *IDS*↑, *COASY*↓, *SULF2*↓ | 0.02 |
| Propanoate Metabolism | Decreased 4/126 (3%). No change 44/126 (35%). Increased 3/126 (2%). No overlap 75/126 (60%). | *LDHAL6A*↓, *DHCR24*↓, *ACSS2*↓, *ACSS1*↑, *LDHAL6B*↓, *LDHB*↑, *ALDH6A1*↑ | 0.02 |
| PPARα/RXRα Activation | Decreased 7/185 (4%). No change 127/185 (69%). Increased 7/185 (4%). No overlap 44/185 (24%). | *MAP2K7*↑, *MED23*↑, *CYP2C9*↓, *RRAS*↑, *TGFBR3*↑, *CREBBP*↓, *ACVR1*↑, *KRAS*↓, *MAPK12*↓, *MED12*↓, *CAND1*↑, *GNAS*↓, *PRKAG2*↑, *PPARGC1A*↑ | 0.03 |
| Sphingolipid Metabolism | Decreased 3/92 (3%). No change 44/92 (48%). Increased 4/92 (4%). No overlap 41/92 (45%). | *SULF1*↑, *SGPP2*↑, *LASS5*↓, *LASS1*↓, *SPHK1*↑, *SMPD1*↑, *SULF2*↓ | 0.03 |
| PPAR Signaling | Decreased 5/101 (5%). No change 68/101 (67%). Increased 4/101 (4%). No overlap 24/101 (24%). | *TRAF2*↑, *RRAS*↑, *CREBBP*↓, *KRAS*↓, *IL1F7*↓, *TNFRSF1B*↓, *PDGFD*↑, *MAPK12*↓, *PPARGC1A*↑ | 0.03 |
| IL-6 Signaling | Decreased 5/96 (5%). No change 75/96 (78%). Increased 4/96 (4%). No overlap 12/96 (13%). | *IL8*↑, *MAP2K7*↑, *TRAF2*↑, *RRAS*↑, *KRAS*↓, *LBP*↓, *IL1F7*↓, *TNFRSF1B*↓, *MAPK12*↓ | 0.04 |
| Pyruvate Metabolism | Decreased 3/145 (2%). No change 51/145 (35%). Increased 4/145 (3%). No overlap 87/145 (60%). | *NKD1*↑, *LDHAL6A*↓, *ACSS2*↓, *ACSS1*↑, *LDHAL6B*↓, *HAGH*↑, *LDHB*↑ | 0.04 |
| Hepatic Fibrosis / Hepatic Stellate Cell Activation | Decreased 8/135 (6%). No change 101/135 (75%). Increased 3/135 (2%). No overlap 23/135 (17%). | *MYL9*↓, *IL8*↑, *EDNRB*↓, *FGF2*↑, *CSF1*↑, *IGFBP3*↓, *LBP*↓, *TNFRSF1B*↓, *MYL3*↓, *FASLG*↓, *MYL7*↓ | <0.05 |
| **Subcutaneous adipose tissue** | | | |
| **Pathway** | **Regulation in type 2 diabetes** | **Genes** | ***P*** |
| Fructose and Mannose Metabolism | Decreased 9/140 (6%). No change 31/140 (22%). Increased 0/140 (0%). No overlap 100/140 (71%). | *GMPPA*↓, *KHK*↓, *HKDC1*↓, *PFKP*↓, *HK3*↓, *FUT7*↓, *FUK*↓, *PFKFB2*↓, *MPI*↓ | 0.01 |
| Circadian Rhythm Signaling | Decreased 4/32 (13%). No change 20/32 (63%). Increased 2/32 (6%). No overlap 6/32 (19%). | *CSNK1E*↑, *AVP*↓, *GRIN2A*↓, *BHLHB2*↑, *CRY2*↓, *CREB3*↓ | 0.04 |
